# Strain Evolution in Cold-Warm Forged Steel Components Studied by Means of EBSD Technique

**DOI:** 10.3390/ma10121441

**Published:** 2017-12-18

**Authors:** Paolo Ferro, Franco Bonollo, Fabio Bassan, Filippo Berto

**Affiliations:** 1 Department of Management and Engineering, University of Padua, I-36100 Vicenza, Italy; bonollo@gest.unipd.it; 2 Zoppelletto S.p.A., Via Camisana, I-36040 Vicenza, Italy; fabio.bassan@zoppelletto.it; 3 Department of Engineering Design and Materials, NTNU, Richard Birkelands vei 2b, 7491 Trondheim, Norway; filippo.berto@ntnu.no

**Keywords:** low-carbon steel, stainless steel, EBSD, cold and warm forging

## Abstract

Electron BackScatter Diffraction (EBSD) in conjunction with Field-Emission Environmental Scanning Electron Microscopy (FEG-ESEM) has been used to evaluate the microstructural and local plastic strain evolution in different alloys (AISI 1005, AISI 304L and Duplex 2205) deformed by a single-stage cold and warm forging process. The present work is aimed to describe the different behavior of the austenite and ferrite during plastic deformation as a function of different forging temperatures. Several topological EBSD maps have been measured on the deformed and undeformed states. Then, image quality factor, distributions of the grain size and misorientation have been analyzed in detail. In the austenitic stainless steel, the γ-phase has been found to harden more easily, then α-phase and γ-phase in AISI 1005 and in duplex stainless steel, sequentially. Compared to the high fraction of continuous dynamic recrystallized austenitic zones observed in stainless steels samples forged at low temperatures, the austenitic microstructure of samples forged at higher temperatures, 600–700 °C, has been found to be mainly characterized by large and elongated grains with some colonies of fine nearly-equiaxed grains attributed to discontinuous dynamic recrystallization.

## 1. Introduction

Properties of metallic materials depend significantly on their microstructure. Two of the most important parameters affecting mechanical behavior of metals and alloys are the grain size and the strain hardening. During forging processes, interconnected variables, such as strain, strain rate, strain distributions and temperature, control the microstructure evolution. Altan et al. [1] indicated the importance of deformation temperature by stating that, above the recrystallization temperature of a formed metal, strain rate is the significant processing parameter, while, below the recrystallization temperature, strain is the processing parameter of primary importance. Herzberg [2] defined metal deformation above the recrystallization temperature as hot-working. McQueen [3] revealed that for many metals there is also a transitional region of forming temperatures between hot working and cold working, within which both strain and strain rate, as well as deformation temperature, interact to affect the resulting microstructure and mechanical properties. This intermediate temperature range is often called warm working range.

Recently, forging producers are increasingly using precision forging, in which complicated parts can be formed directly in net shape or near-net shape, to reduce costs and save time. Particularly, in cold forging, materials with high formability are required. Low carbon steels are widely used since they are less crack-sensitive during forging operations [4].

In this scenario, stainless steels are an important class of alloys. Their importance is manifested in the plenitude of applications that rely on their use. The application of austenitic stainless steels in food, petrochemical and nuclear industries is due to their combination of mechanical and corrosion resistance. In particular, AISI 304L steel is widely used, not only for its high corrosion resistance but also for its excellent formability and mechanical behavior. Many researchers have studied the changes in 304L stainless steel. Its static plastic deformation and corresponding microstructural evolution was found different from dynamic loading conditions at high strain rate [5,6,7,8,9]. Another steel grade of great interest for forging industry is the Duplex Stainless Steel (DSS). DSS is a two-phase alloy (ferrite/austenite) which combines the properties of austenitic and ferritic stainless steels. The good combination of its mechanical properties and corrosion resistance makes it of great interest for a wide range of applications especially in the oil, chemical and power industry [10]. During the last years, in view of the great interest of forging industries on these materials, several studies on their formability were conducted. It is noted that its properties strongly depend on the microstructure and substructural changes of α- and γ-phase during deformation under low and high strain rate conditions [11,12].

Grain boundary character plays a key role in the plastic deformation of polycrystalline materials and a beneficial combination of mechanical properties can be achieved by grain refinement. In particular, the mechanical properties of carbon and stainless steels can be improved by fine-grained structures [13,14,15,16]. Such materials do not undergo phase transformations within a wide temperature range, and small grain sizes can be produced by dynamic recrystallization (DRX) under warm or cold forging conditions [17,18]. Since size of the dynamically recrystallized grain sensitively depends on processing temperature, the fine-grained microstructures can be developed under warm deformation conditions, i.e., during plastic working at relatively low temperature (T = *0.5–0.7 T_m_ with T_m_ the melting temperature*) [19]. Recently, two main DRX mechanisms have been found to operate in metallic materials with low stacking fault energy (SFE): discontinuous DRX (DDRX) and continuous DRX (CDRX). In the DDRX mechanism, the formation of a new grain structure results from the operation of a grain boundary bulging, namely grain boundary serration and migration consuming the strain hardened substructures [20]. The recrystallized structure can be achieved by using conventional metal working techniques consisting in recrystallized and work hardened component [15,21,22].

The other type is the continuous DRX, which operates mainly under conditions of warm working [23]. The new grains develop as a result of the gradual increase in the misorientations between the subgrains that are caused by the plastic deformation; thus, fine-grained materials cannot be produced by standard thermomechanical processing [17,19,20].

The present work is aimed to describe qualitatively and quantitatively the differences in the plastic behavior of ferrite and austenite during one-stage cold forging process to form a hex-head plug fitting used in thermo-hydraulic applications. The strain heterogeneities and microstructural evolution of γ-phase in AISI 304L and Duplex 2205 stainless steel during warm forging process at different temperatures (i.e., 400, 500, 600 and 700 °C) are also investigated. Finally, the strain hardening behavior of the steels at cold and warm working conditions is fully analyzed.

## 2. Materials and Methods

The chemical composition of the alloys analyzed (AISI 1005 (Wr. N. 1.0303), AISI 304L (Wr. N. 1.4307), and DDS 2205 (Wr. N. 1.4462)) are listed in Table 1.

In the as-received conditions, the materials were obtained by continuous casting and then hot rolled down to a final bar diameter of 22 mm. AISI 304L and DDS 2205 steel bars were solution heat-treated at 1150 °C and 1050 °C, respectively, and water-quenched to avoid precipitation of secondary phases.

**Figure 1:**
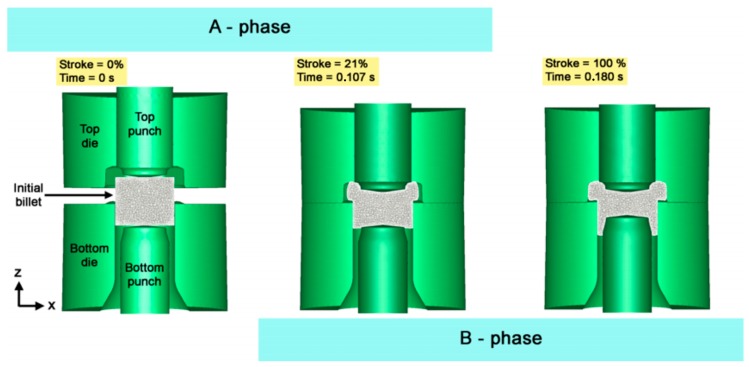
Set-up of the single-stage forging test.

Figure 1 shows the step-sequence of the analyzed one-stage forging process at different strokes. The process consisted of two forging phases: first, a compression to create the hex-head (named “A-phase”); and, second, a deep backward extrusion operation to form the “neck” of the plug fitting (named “B-phase”). Bottom punch was fixed during the forming cycle. Top punch and die were driven by press mechanism. Moreover, bottom die was floating and driven by the contact forces.

3D solid modeling of the workpiece (i.e., cylindrical billet, 18.3 mm height and 55 g weight) and tools were carried out by Pro/E^®^ (Needham, MA, USA) software and then imported into FORGE2011^®^ (Mougins, France) numerical code. 

Details about the numerical models such as materials rheology and friction conditions can be found in previous works [24,25]. In total, 550 cylindrical billets (50 in AISI 1005, 250 in AISI 304L and 250 in Duplex 2205) were used for cold and warm forging experimental tests. Samples were forged by using a 2453 kN single-station general-purpose mechanical knuckle press with 50 spm (stroke per minute). 

**Figure 2:**
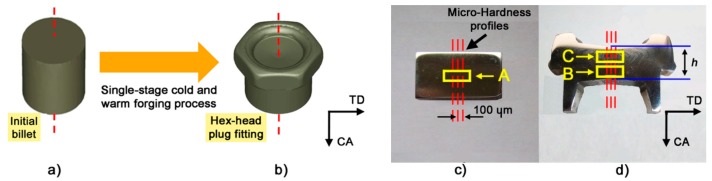
CAD geometry of: initial billet (**a**); and hex-head plug (**b**); and longitudinal sections of: real billet (**c**); and forged component (**d**).

For a detailed understanding of the effects caused by cold and warm forging processes on the alloy, metallographic longitudinal sections parallel to the compression *z-axis* (CA) were drawn from the cylindrical billets and forged samples at different temperatures (Figure 2). The focus was set on the microstructural analysis of three areas corresponding to different strain levels, named *zone A* (no deformation), *zone B* (intermediate level of strain) and *zone C* (high strain level). The boundary between *zones C* and *B* was chosen, according to the numerical simulation, equal to effective strain of 0.6 (Figure 5). Height reductions h (Figure 2d), defined as the ratio between the height of the deformed part (highlighted in Figure 2d) and the height of the initial billet before forging operations (Figure 2a), for each alloy at different forging temperatures are reported in Table 2. 

For optical investigations and micro-hardness measurements, AISI 1005 specimens were etched with 4% HNO_3_ in ethanolic solution; AISI 304L was etched with a reagent for electrolytic etching (a mixture of 60% HNO_3_ and 40% distilled water); and Duplex 2205 samples were etched with Beraha etching solution (10 mL HCl, 40 mL distilled water, 1 g K_2_S_2_O_5_). The micro-hardness tester Vickers Leitz Wetzlar D-35578 (Leica, Wetzlar, Germany) was used to perform three micro-hardness profiles, as shown in Figure 2c,d. Measurements were carried out according to Standards ASTM E92-82 using a load of 100 g. Microstructural investigation was also carried out by using a FEI Quanta 250 scanning electron microscope (FEI, Hillsboro, OR, USA) equipped with an electron back scattering diffraction (EBSD) analyzer incorporating an orientation imaging microscopy (OIM) system (EDAX TSL software, version 5). The surfaces of the undeformed and cold-warm forged specimens were prepared by using a polishing solution of 0.05 μm colloidal silica suspension and then electropolished in an electrolytic etching solution (60 mL HClO_4_, 40 mL distilled water) at 20 °C to ensure the highest surface quality. Samples were placed in FEG-ESEM microscope (FEI, Hillsboro, OR, USA) immediately after preparation. To compare the strain levels of *zone A* and *B*, step and area size used in the EBSD scans were 50 nm and 300 × 300 μm^2^ respectively; on the other hand, because of the different strain levels between *zone B* and C and thus different quality of the scanning micrographs, the comparison between *zone A* and *B* was made by using a step and area size of 70 nm and 150 × 150 μm^2^, respectively. The OIM images were subjected to clean-up procedures by setting a minimal confident index of 0.1. For the EBSD analysis a working distance in the range of 13–21 mm, a voltage eqaul to 20 kV, a beam current of 220 μA, an fps (frame per second) of 30 and a number of maps per zone equal to 3 were chosen.

## 3. Results and Discussion

### 3.1. Optical Microscope Observations (Forging Temperature, 20 °C)

**Figure 3:**
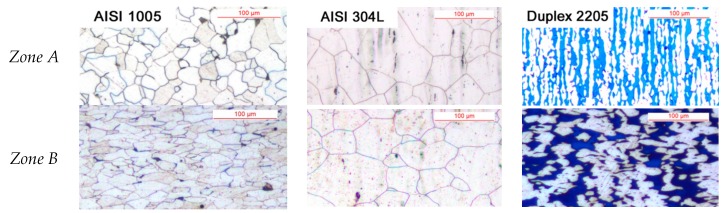

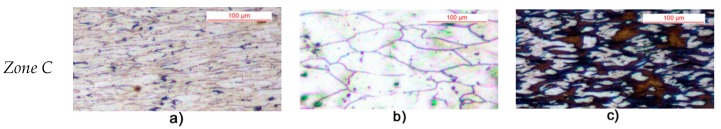
Microstructure of: (**a**) AISI 1005; (**b**) AISI 304L; and (**c**) DDS 2205, as a function of the analyzed zone (Figure 2c,d).

Figure 3 shows the alloys microstructures before and after the cold forging test. In the as-received state (Figure 3a—*zone A*), AISI 1005 is characterized by a ferritic microstructure with a low amount of pearlite and an average grain size of 21 μm; its hardness value was found to be equal to 128 ± 3 HV_0.1_. AISI 304L (Figure 3b—*zone A*) shows the typical austenitic microstructure with twin boundaries; initial values of average grain size and hardness were found to be 42 μm and 207 ± 4 HV_0.1_, respectively. 

Finally, the grain size and hardness of the ferritic-austenitic stainless steel (DDS 2205, Figure 3c—*zone A*) were 9 μm and 245 ± 6 HV_0.1_, respectively. In the as-received state, a balanced amount of austenite-ferrite was observed. 

### 3.2. Micro-Hardness Evolution (Forging Temperature, 20 °C)

**Figure 4:**
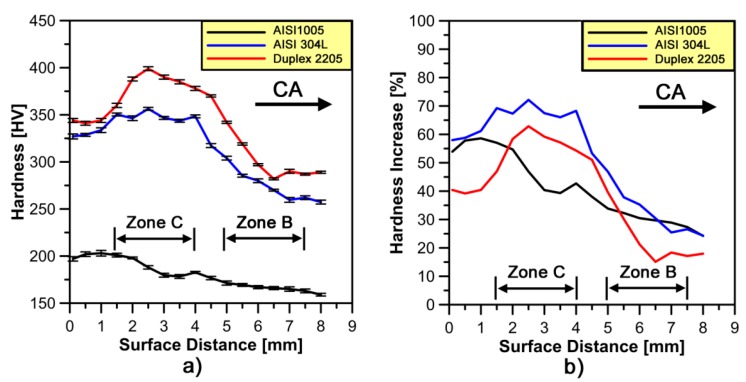
Average: (**a**) micro-hardness profiles; and (**b**) trends of micro-hardness increase rate as a function of distance along the compression axis (CA). Data refer to the investigated steels.

Micro-hardness profiles reveal different hardening intensities for cold forged tested steels (Figure 4a). AISI 1005, due to the almost full presence of α-phase, shows a nearly homogeneous hardening behavior. The highest mean values of hardness can be observed on the area close to the contact surface between the top punch and the workpiece (Figure 4a). This can be correlated to the combination of the material elastic-plastic properties (low stain hardening coefficient) and the forging technique used.

**Figure 5:**
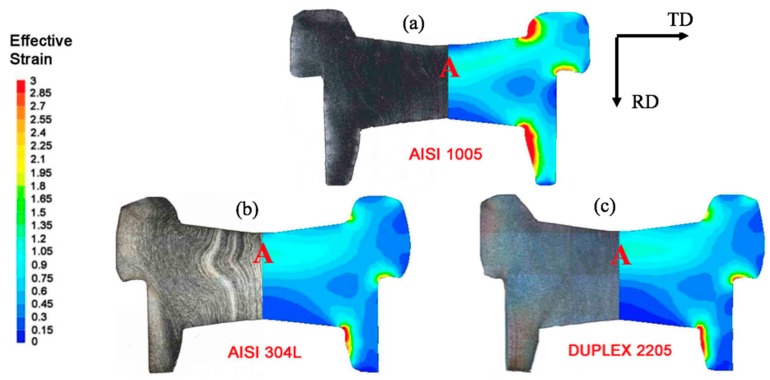
Effective strain obtained by FE analysis (**Right** side) and macrographs of sample cross section (**Left** side). AISI 1005 (**a**); AISI 304L (**b**); DUPLEX 2205 (**c**).

The cold forged stainless steel samples show an inhomogeneous hardening behavior with a hardness increase in *zone C* (Figure 4a). For AISI 304L, the hardness values vary from 356 to 257 HV_0.1_; while, in the case of DSS, they are in the range of 399 to 282 HV_0.1_. The highest hardness properties of the ferritic-austenitic stainless steel is mainly associated to the higher mean values of hardness reached on the as-received state ((~245 ± 6) HV_0.1_). Furthermore, due to the higher strain hardening coefficients of stainless steels compared to low carbon steel, the deformation tends to localize in *zone C* forming a sort of barrier that prevents the material flow to extend into other zones of the mold (Figure 5). This has been also confirmed by the distribution of the micro-hardness increase $\left({\bar{HV}}_{0.1}\left(\%\right)\right)$ (Figure 4b) defined as:

(1) $${\bar{HV}}_{0.1}\left[\%\right]=\frac{{\bar{HV}}_{0.1\;\left(deformed\right)}-{\bar{HV}}_{0.1\;\left(as-received\right)}}{{\bar{HV}}_{0.1\;\left(as-received\right)}}\;$$

where ${\bar{HV}}_{0.1\;\left(deformed\right)}$ and ${\bar{HV}}_{0.1\;\left(as-received\right)}$ are the mean values of micro-hardness derived from the three profiles reported in Figure 2c,d respectively, calculated at the same distance along the compression axis (CA). It is easy to show that stainless steels are characterized by a rapid hardness increase from *zone B* to *zone C*. Even if DSS is characterized by a higher hardness value in the as-received conditions, which makes it more difficult to forge, it has a lower tendency to harden than AISI 304L steel (Figure 4b). The highest strain hardening effect of the fully austenitic stainless steel is associated to the twin boundaries formation [26], crossing of slip planes [27], the increase of dislocation and stacking fault density in the deformed regions [28,29].

### 3.3. Electron Backscatter Diffraction Analysis (Forging Temperature, 20 °C)

Several statistical analyses have been performed on the EBSD data from each scanned area (*zone A–C*) to compare local plastic strain and grain evolution behavior of α- and γ-phase on the as-received and deformed steels considered.

#### 3.3.1. Image Quality (IQ) Factor

**Figure 6:**
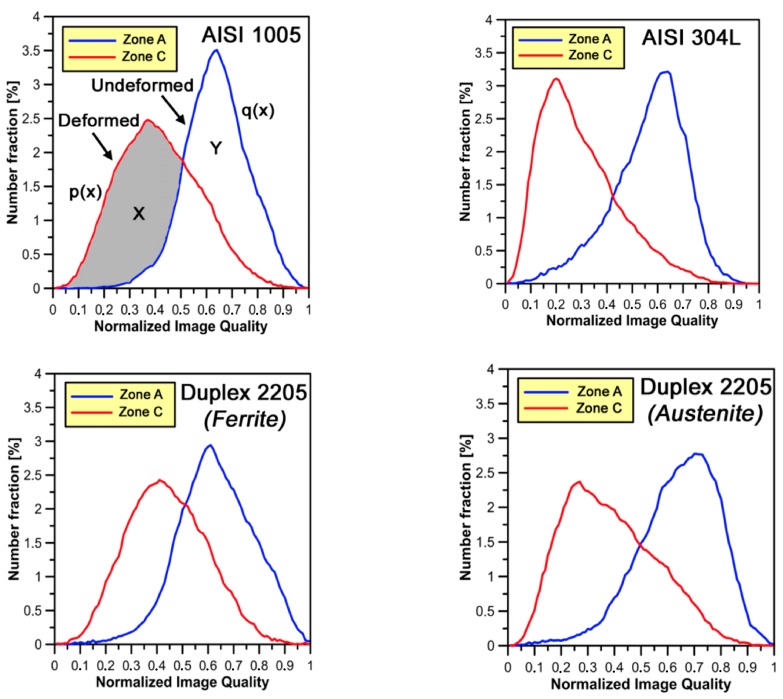
Normalized IQ distribution for various degrees of strain at 20 °C for the two phases on the different steels investigated.

At each measurement point in an OIM scan, a parameter quantifying the quality of the corresponding diffraction pattern is recorded. It is well known [30] that the image quality (IQ) is affected by residual strain in the diffracting volume. Thus, an indication of the distribution of strain in the material can be observed through an IQ map. For a large scanned area on a bulk sample, if the average IQ value is assumed to correspond to the overall strain measured mechanically, the local strain can be quantified by assuming a linear relationship between the IQ value and the local plastic strain. In this work, the quantitative evaluation method of the local plastic strain rate is based on the concept proposed by Tarasiuk et al. [31]. The idea is reported in Figure 6. In each graph, two normalized IQ distributions are plotted which correspond to the undeformed and deformed sample. The total area under each distribution curve is equal to one since it includes all the points within the area under investigation used to estimate deformed and undeformed material volume fractions. By superposing these two plots, two areas are detected: *region X*, which corresponds to all the points deformed without ambiguity, and *region Y*, which corresponds to the still undeformed points (Figure 6). The area of *region X* is used to estimate the minimal deformed volume fraction (*V_f min_*) according to the following equation (Equation (2)):

(2) $$V_{f\;min}=\overset{}{\underset{x:p\left(x\right)>q\left(x\right)}{\int }}\left[p\left(x\right)-q\left(x\right)\right]dx\;$$

where *p*(*x*) and *q*(*x*) are the normalized IQ distributions for deformed and undeformed samples, respectively. 

Figure 6 shows the IQ normalized distributions as a function of phase (ferrite, austenite) corresponding to the as-received (*zone A*) and cold forged (i.e., *zone C*) materials (the IQ distributions measured in *zone B* are directly used in the subsequent calculations of *V_f min_* fraction). For AISI 304L, the absolute IQ ranges were 250–1500 (*zone C*) and 250–750 (*zone A*). Similar value ranges have been obtained for AISI 1005 and Duplex 2205. In both phases, plastic strain leads to a shift of the normalized IQ distribution peak to lower values.

In Table 3, the *V_f min_* fraction is estimated (Equation (2)) by assuming that the as-received state of steels is deformation free (*V_f min_ =* 0%).

In Table 3, it can be easily noted that the highest values of *V_f min_* fraction are reached on γ-phase. This is confirmed by the average misorientation angles determined by EBSD that has been observed (in the present work and in a previous study [32]) to increase faster in γ-phase than in α-phase.

Variations of 39% to 44% in α-phase for AISI 1005 and Duplex 2205 steel, and of 46% to 50% in γ-phase for AISI 304L and DDS 2205 are observed; the difference is attributed to the slightly higher tendency to work-harden of single-phase steels (AISI 304L) as mentioned above. Moreover, DDS 2205 shows the highest values of *V_f min_* fraction for both phases in *zone C*. This may be correlated with the highest micro-hardness values previously measured on that area.

#### 3.3.2. Microstructural Evolution (Forging Temperature, 20 °C)

**Figure 7:**
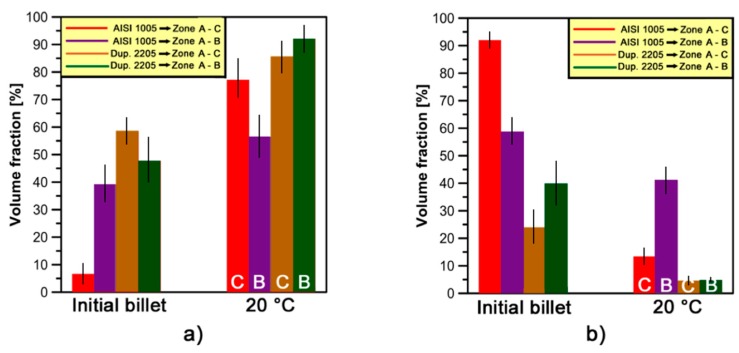
Volume fractions of: (**a**) LABs; and (**b**) HABs developed in α-phase for AISI 1005 and DDS 2205 samples at 20 °C and different strain levels.

**Figure 8:**
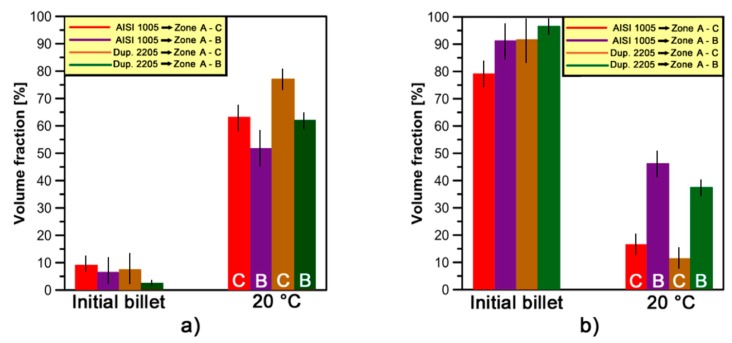
Volume fractions of: (**a**) LABs; and (**b**) HABs developed in γ-phase for AISI 304L and DDS 2205 samples at 20 °C and different strain levels.

A detailed analysis of the misorientation angle distributions by EBSD with the aim to estimate the amount of low-angle boundaries (LABs) (θ = 2°–5°) [33] and high-angle boundaries (HABs) (θ > 15°) [34] has been carried out. Figure 7 and Figure 8 show the histograms of LABs and HABs volume fractions (defined as the ratio between LABs length (or HABs) and the total grain boundary length, mm/mm * 100) as a function of the analyzed zone. 

It can be noted that the volume fraction of LABs increases and HABs decreases after a cold forging cycle in both phases. This can be attributed to the development of a sub-grains microstructure, characterized by dislocation walls, which forms during plastic deformation.

With the exception of AISI 1005, a higher amount of LABs has been detected in the α-phase compared to γ-phase in the as-received state. This upholds the hypothesis of incomplete recrystallization of α-phase. On the other hand, HABs prevail in γ-phase. They form through the fragmentation of elongated grains, as provided after complete recrystallization. The high difference among initial LABs and HABs volume fraction values of AISI 1005 was due to the supply conditions and the presence of a single-phase microstructure.

**Figure 9:**
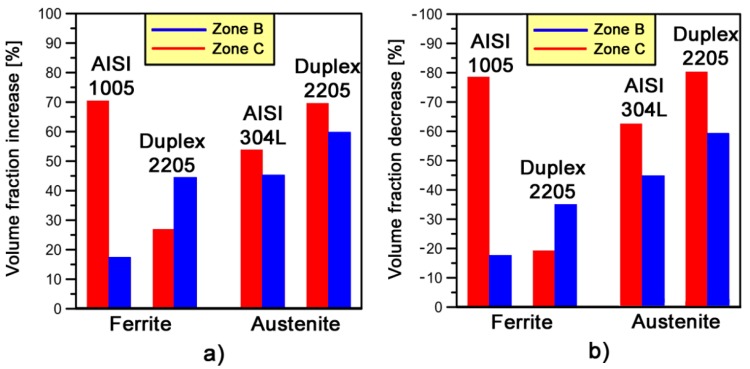
(**a**) LABs volume fractions increase; and (**b**) HABs volume fractions decrease on *zones B* and *C* for both phases.

The variation of LAB and HAB fraction within a phase may depend on different parameters. In Figure 9, the LABs increase and HABs decrease is calculated as difference between the associated deformed (*zones B* and *C*) and undeformed (i.e., *zone A*) values reported in Figure 7 and Figure 8.

In Figure 8a, it can be observed that the highest increase of LABs volume fractions has been found on the α-phase of low carbon steel (*zone C*). This is due to the higher formability properties of this material compared to the stainless steels analyzed. It is also in good agreement with the high *V_f min_* increase estimated on that phase for AISI 1005 (Table 3). On the other hand, the LABs fraction increase in *zone B* is higher for DDS 2205 compared to AISI 1005 steel as a consequence of the higher *V_f min_* fraction value reached in that zone.

The increase of LABs volume fractions in γ-phase, on both deformed zones (~20–25%), is higher in DSS 2205 than AISI 304L. This is due to the building up of higher amount of dislocation microstructure composed by sub-grains induced by the higher levels of strain (*V_f min_* fraction) observed on γ-phase of DDS 2205 compared to AISI 304L. Moreover, the highest increase of LABs volume fractions observed on *zone C* (high strain area) for the stainless steels is directly related to their high strain-hardening behavior (Figure 4b). A very similar histogram is observed for the HABs volume fractions decrease (Figure 8b).

A detailed statistical analysis of the misorientation distribution angles across the so-called special γ-grain boundaries, i.e., those having dense Coincident Site Lattice (*CSL*), was also carried out. By using EBSD analysis, the CSL numbers (Σ) were measured by means of the following equation:

(3) $$\Sigma =\frac{number\;of\;lattice\;points\;in\;the\;unit\;cell\;of\;a\;CSL}{number\;\mathrm{of}\;\mathrm{lattice}\;\mathrm{points}\;\mathrm{in}\;a\;\mathrm{unit}\;\mathrm{cell}\;\mathrm{of}\;\mathrm{the}\;\mathrm{generating}\;\mathrm{lattice}}\;$$

In face centered cubic metals and alloys with low stacking fault energy (SFE), most of these special boundaries are Ʃ3 or Ʃ3^n^ CSL boundaries related to twin boundaries. On *zone A*, about 59.2% and 65.9% of the HABs on AISI 304L and DSS 2205, respectively, display the first-order twin CSL orientation relationship Ʃ3 (within a deviation of 2°) characterized by 60° rotation about <111> axis. About 3.2% of boundaries in AISI 304L and 3.0% of boundaries in DDS 2205 appear to correspond to the second-order twins represented by Ʃ9 (38.9°/<011>) CSL orientation relationship.

On *zone B*, the γ-phase regions become slightly more elongated and locally fragmented compared to *zone A*. They show a tendency to become preferentially aligned at determined angles. The originally sharp peak in the γ-phase misorientation distribution centered on the ideal Ʃ3 CSL orientation relationship becomes broader and the portion of first-order twin boundaries among the HABs decrease to about 55.8% and 64.7% on AISI 304L and Duplex 2205 steel, respectively.

As the plastic strain increases (i.e., *zone C*), the austenite areas become more elongated. The broadening of the original Ʃ3 peak in the γ-phase misorientation distribution becomes more pronounced; the portion of the first-order twin boundaries among the HABs further decreases to about 14.3% and 12.3% on fully austenitic stainless steel and DDS 2205, respectively. The observed presence of second-order twin boundaries in the misorientation spectra is about 1.1% for AISI 304L and 2.0% for the duplex stainless steel. Table 4 summarizes the main fractions of CSL boundaries examined in the γ-phase; α-phase is practically free of them.

The present results show that pre-existing annealing twin regions within the austenite display a tendency to progressively rotate away from the ideal CSL orientation relationship during straining. 

Thus, the corresponding originally straight coherent twin boundaries become gradually converted to general HABs during the deformation process. Similar results showing that such rotations appear to occur very early in the deformation process and might reach values of several tens of degrees at large strains have been reported by Cizek et al. [35]. 

## 4. Effect of Temperature on Microstructural Evolution of γ-phase

### 4.1. Micro-Hardness Evolution on Zones B and C at Different Warm Forging Temperatures

**Figure 10:**
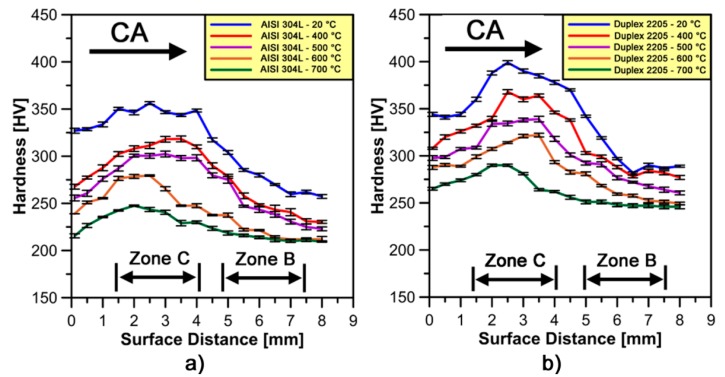
Average micro-hardness profiles as a function of distance along the compression axis (CA) for: (**a**) AISI 304L; and (**b**) Duplex 2205 stainless steel.

Figure 10a,b shows micro-hardness profiles on stainless steel samples forged at different temperatures. The effect of increasing temperature tends to continuously decrease the micro-hardness profiles on each type of steel. This behavior is mainly associated with the higher dislocation mobility and lower dislocation density at higher forging temperatures [22]. All micro-hardness profiles confirm the presence of the high strain-hardened area around *zone C* under the forging impact at different temperatures. This effect is less pronounced at higher forging temperatures tests.

**Figure 11:**
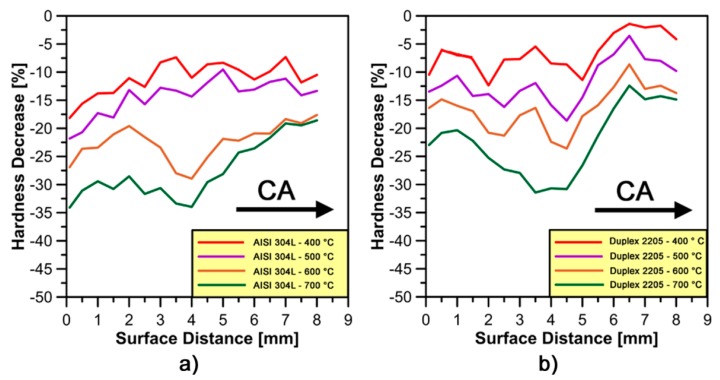
Average trends of microhardness decrease rate as a function of distance along the compression axis (CA) for: (**a**) AISI 304L; and (**b**) Duplex 2205 stainless steel.

In Figure 11a,b, a comparison between micro-hardness profiles obtained at 20 °C and at different forging temperatures has been made on each stainless steel in terms of micro-hardness decrease rate measurements $\left({\bar{HV}}_{0.1}\;\left[\%\right]\right)$ estimated by Equation (1).

As shown in Figure 11b, Duplex 2205 has a lower tendency to decrease micro-hardness values at different forging temperatures than AISI 304L; moreover, the increase of temperature seems to drastically decrease the strain hardening effect in *zone C*. This behavior is not clear on AISI 304L samples forged at 400 and 500 °C, respectively, due to lower temperatures and similar micro-hardness profiles as compared to cold forged material.

### 4.2. Electron Backscatter Diffraction Analysis of Zones B and C at Different Warm Forging Temperatures

#### 4.2.1. Minimum Deformed Volume Fractions

**Figure 12:**
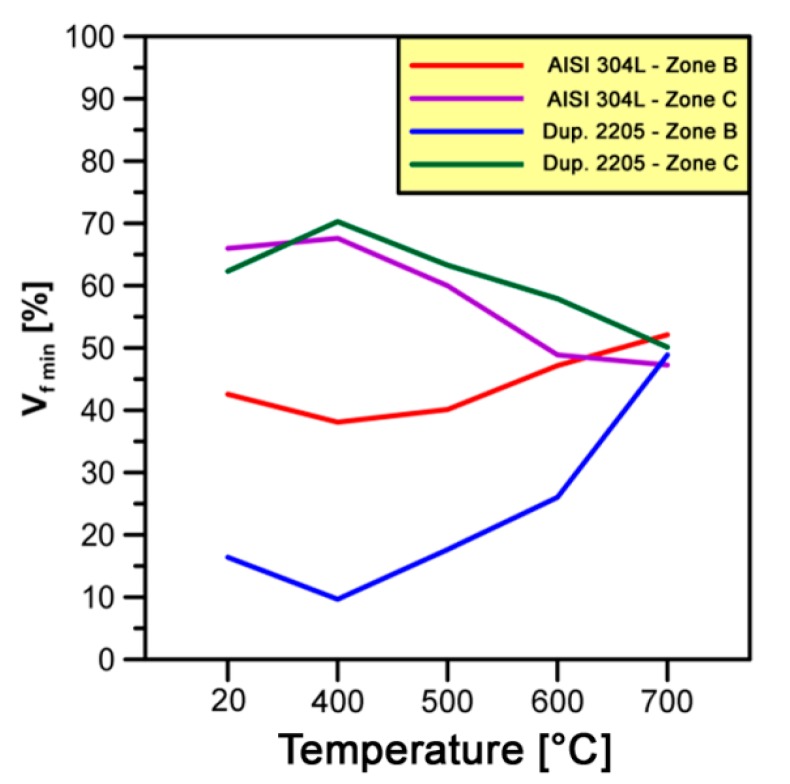
Relationships between the minimum deformed volume fractions (*V_f min_*) and forging temperatures evolved under cold-to-warm deformation of γ-phase in AISI 304L and Duplex 2205 stainless steel.

Figure 12 shows the trends of *V_f min_* fractions for AISI 304L and Duplex 2205 stainless steel at different forging temperatures.

As can be seen in Figure 12, the *V_f min_* fractions are almost constant with an increase in forging temperature from 20 to 400 °C on *zone C* in both steels analyzed; they slightly decrease on *zone B*. The warm-working temperature of 400 °C gives not enough relevant alteration and evolution on γ-grains and sub-grains structures at different levels of strain (i.e., *zones B* and *C*). In *zone B*, with an increase in temperature from 400 to 700 °C, the *V_f min_* fractions are in the range of 38–52% and 10–49% in AISI 304L and DSS 2205, respectively. On the other hand, in the same range of temperatures, the *V_f min_* fractions vary from 47% to 68% and from 50% to 70% in the austenitic and dual-phase stainless steel, respectively. If this range of temperatures is considered, variations of 14% and 39% in *V_f min_* fractions are observed in *zone B* for AISI 304L and Duplex 2205, respectively. On the other hand, the raising of *V_f min_* fractions are almost constant and set at about 20% on *zone C* for both steels; it means that the γ-phase presents the same formability properties at different temperatures in both steels.

In *zone B*, the different increase of *V_f min_* fractions on the two steels analyzed is a direct consequence of the forging process used. *Zone B* is the last zone of the workpiece to be deformed and the lowest formability properties DDS 2205 steel involve lower *V_f min_* fractions at low temperatures as compared to AISI 304L steel. Due to the temperature increase effect, a reduction of *V_f min_* fractions gap between the steels analyzed is revealed and the formability properties of Duplex 2205 are greatly improved. In fact, in Figure 12, it is noted that γ-phase reaches almost similar *V_f min_* fractions at 700 °C in both steels considered.

#### 4.2.2. Microstructural Evolution of γ-phase on Zones B and C at Different Warm Forging Temperatures

**Figure 13:**
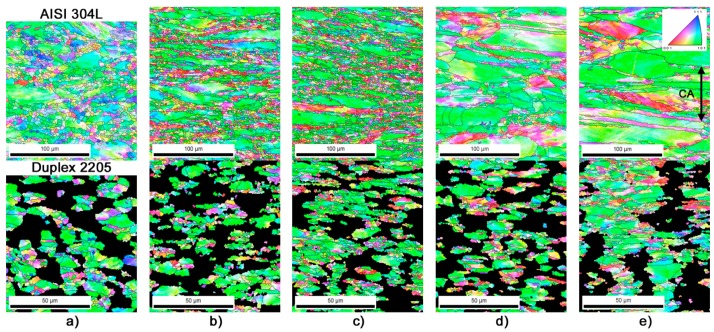
OIM micrographs on *zone C* for deformed γ-phase microstructure evolved in the AISI 304L and DDS 2205 steel processed by one-stage forging process at: (**a**) 20 °C; (**b**) 400 °C; (**c**) 500 °C; (**d**) 600 °C; and (**e**) 700 °C. The inverse pole figure is shown for the compression *z-axis* (CA). The black lines indicate high-angle boundaries (HABs) while the big black areas correspond to the missed ferrite grains.

The deformation microstructures obtained after one-stage forging process at temperatures of 20 °C, 400 °C, 500 °C, 600 °C and 700 °C are shown in Figure 13 (black regions in the maps of Duplex 2205 are used for highlighting only the of γ-phase). 

**Figure 14:**
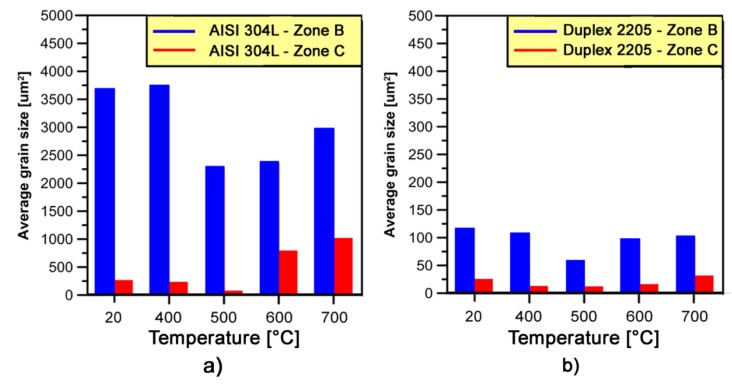
Average γ-grain size evolved in the: (a) AISI 304L; and (b) Duplex 2205 stainless steel after forming process on *zones B* and *C* at different forging temperatures (each value is the result of at least 20 OIM maps without taking into account grains at the edges).

The single-stage warm forging process at all studied zones (i.e., *Zones B* and *C*) and temperatures results in slight γ-grain refinement from 20 to 500 °C for each stainless steel considered. A non-uniform fine grained structure evolves in the samples processed at these temperatures in *zone B* and *C*, as reported in Figure 14. The γ-grains size of AISI 304L (forging temperature from 20 to 500 °C) are in the range of 3671–2285 μm^2^ and 267–77 μm^2^ in *zones B* and *C*, respectively. In the same temperature range, the average γ-grains size of DDS 2205 changes from 116 to 58 μm^2^ in *zone B* and from 25 to 12 μm^2^ in *zone C*. The microstructure at these temperatures is characterized by some heterogeneities. In addition to the equiaxed fine dynamic recrystallized (DRX) grains, these microstructures contain a low amount of large-elongated γ-grains with irregular boundaries, which are the remainders of the original grains.

At 600 and 700 °C the microstructures are almost fully composed of fine nearly-equiaxed and large-elongated γ-grains. No significant DRX took place at those temperatures. Since γ-phase is characterized by a low value of stacking fault energy, the partially recrystallized microstructure at 600 and 700 °C is attributed to DDRX [36,37].

**Figure 15:**
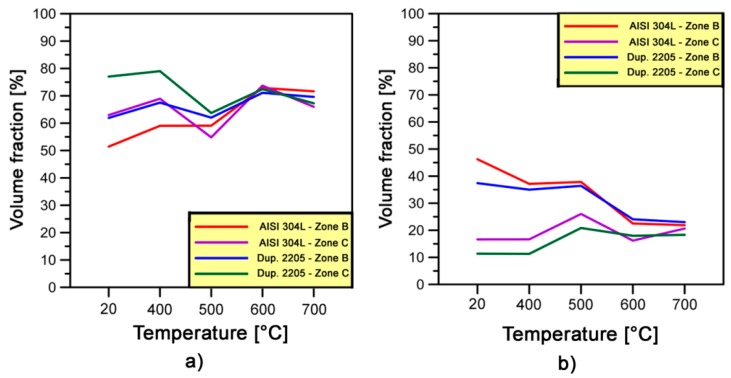
Fractions of: (**a**) LABs; and (**b**) HABs estimated in both stainless steels at different forging temperatures.

Figure 15 shows an examination of the boundary and sub-boundary misorientation distribution during deformation process at different forging temperatures. The fractions of LABs (i.e., 2 ≤ θ ≤ 5°) and HABs (i.e., 15° ≤ θ ≤ 90°) tend to become almost similar at higher temperatures (i.e., 600 and 700 °C) for both deformed zones and stainless steels considered. The originally large differences between fractions of LABs on both deformed zones at 20 °C (i.e., ~12% for AISI 304L and 15% for Duplex 2205), decrease remarkably to about 6% and 2% on single-phase and dual-phase steel, respectively, at 700 °C. At this temperature, the fractions of LABs vary slightly from 66% to 72% (Figure 15a). On the other hand, in Figure 15b, a very similar behavior of HABs fractions are also revealed at different forging temperatures. At 20 °C the differences between fractions of HABs on both deformed zones are 30% for AISI 304L steel and 26% for Duplex 2205 steel, which decrease drastically to 1% and 5% on single-phase and dual-phase steel, respectively, at 700 °C.

**Figure 16:**
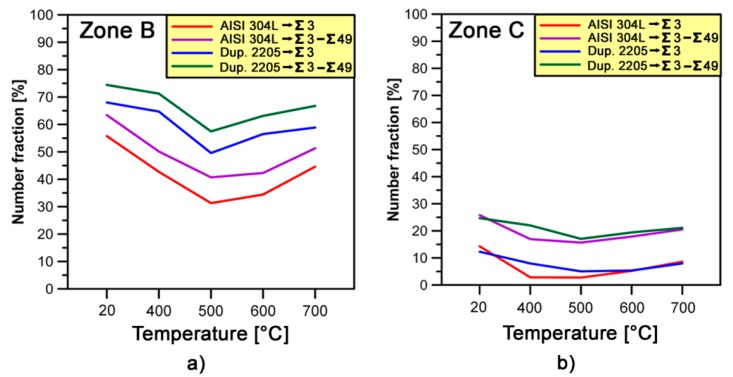
Fractions of CSL boundaries in γ-phase estimated at different forging temperatures: (**a**) *zone A*; and (**b**) *zone C*.

As regards the specific misorientations that might be present within the stainless steels, the fraction of CSL boundaries has also been examined in the γ-phase. Resulting values are graphically shown in Figure 16. At lower temperatures (i.e., from 20 to 500 °C), the fraction of CSL boundaries decreases in both deformed zones of stainless steels analyzed. On *zone B*, Ʃ3 boundaries fraction decreases from 56% to 45% for AISI 304L and from 68% to 59% for DDS 2205. Moreover, it decreases from 14% to 9% and from 25% to 21% for austenitic and dual-phase stainless steel, respectively, on *zone C*. At the same time the percentage of total CSL boundaries decreases simultaneously on both deformed zones of stainless steels. On the other hand, the fractions of CSL boundaries at 600 and 700 °C increase as a consequence of the new grains nucleated as a result of local bulging of grain boundaries during the dynamic recrystallization (DRX) mechanism [38,39].

## 5. Conclusions

Compared to previous works, both cold and warm single-phase forging processes of different steels (AISI 1005, AISI 304L and DDS 2205) were investigated. To the best of authors knowledge, α and γ phases metallurgical behaviors at different temperatures were compared for the first time by means of the same investigation techniques in alloys characterized by the presence of both or one of them. Because of the different microstructure that characterized the alloys analyzed, a different behavior was observed between ferrite and austenite during the cold and warm forging process. Duplex stainless steels are known to be difficult to cold forging. The obtained results give new insights about the cold and warm forging process of the analyzed alloys and above all they show the possibility to cold and warm forging duplex stainless steels. 

The main results can be summarized as follows:

- The α-phase in AISI 1005 steel has a lower tendency to harden compared to γ-phase of AISI 304L steel. On the other hand, γ-phase tends to harden easier on austenitic than on duplex stainless steel. The highest strain hardening effect of γ-phase is associated to the crossing of slip planes, twin boundaries formation, the increase of dislocation and stacking fault density in the deformed regions.
- During the cold forging process, the estimated deformed volume fraction is higher in the γ-phase compared to the α-phase. Furthermore, the γ-phase grains deform more homogeneously than the initially large α-phase grains.
- Samples forged at 20 °C result in the development of fine grained microstructures. Low-angle boundaries (LABs) increase and high-angle boundaries (HABs) decrease, as a direct consequence of the dislocation microstructures formation.
- The γ-phase microstructure which develops during single-stage forging from 400 to 500 °C is characterized by fine grained microstructure at different strain levels. The fraction of special boundaries decreases rapidly from 400 to 500 °C for both stainless steel analyzed. On the other hand, the microstructures of γ-phase detected at higher forging temperatures (i.e., 600 and 700 °C) are almost fully composed of large-elongated and fine nearly-equiaxed grains, which are considered to be discontinuous dynamic recrystallized (DRX). Similar values of LABs and HABs fractions and annealing twins formation are observed on the stainless steels investigated.

## Figures and Tables

**Table 1:** Chemical compositions of the materials analyzed (wt. %).

Steel	C	Si	Mn	Cr	Mo	Ni	Cu	Co	N	Others	Fe
AISI 1005	0.051	0.07	0.30	0.11	0.02	0.13	0.17	0.01	0.01	<0.07	bal.
AISI 304L	0.031	0.35	1.32	18.66	0.40	8.11	0.49	0.12	0.09	<0.06	bal.
Duplex 2205	0.022	0.52	1.28	22.30	3.13	5.68	0.19	0.07	0.18	<0.05	bal.

**Table 2:** Height reductions h at various forging temperatures.

Steel	Height Reduction *h* (%)				
	20 °C	400 °C	500 °C	600 °C	700 °C
AISI 1005	96.1	-	-	-	-
AISI 304L	54.0	56.5	57.4	60.9	64.5
Duplex 2205	49.7	53.2	54.1	57.9	61.0

**Table 3:** *V_f min_* fractions estimated in α and γ-phase after different strain levels.

Investigated Area	*V_f min_* Fraction (%)			
	α-phase (%)		γ-phase (%)	
	AISI 1005	Duplex 2205	AISI 304L	Duplex 2205
*Zone B*	12.3	24.7	14.3	25.8
*Zone C*	55.8	63.4	64.7	71.3

**Table 4:** CSL boundary fractions parameter in the γ-phase.

Investigated Area	CSL Boundaries Volume Fraction (%)			
	AISI 304L		Duplex 2205	
	Ʃ3	Total: Ʃ3–Ʃ49	Ʃ3	Total: Ʃ3–Ʃ49
*Zone A*	59.2	68.1	65.9	73.7
*Zone B*	55.8	63.4	64.7	71.3
*Zone C*	14.3	25.8	12.3	24.7
